# Moral conflicts among patients-caregivers dyads in oncological care pathway: a systematic review of the ethical literature

**DOI:** 10.1007/s00520-025-09548-w

**Published:** 2025-06-06

**Authors:** Clizia Cincidda, Virginia Sanchini, Gabriella Pravettoni

**Affiliations:** 1 Applied Research Division for Cognitive and Psychological Science, European Institute of Oncology IRCCS, Milan, Italy; 2 Department of Oncology and Hemato-Oncology, University of Milan, Via Santa Sofia 9/1, 20122 Milan, Italy; 3 Department of Public Health and Primary Care, Centre for Biomedical Ethics and Law, KU Leuven, Leuven, Belgium

**Keywords:** Cancer, Caregiver, Relationship, Bioethics, Systematic review

## Abstract

**Purpose:**

Cancer patients are most often accompanied by at least one caregiver along the oncological care path. Since cancer has been defined as a “family disease”, patients may rely on caregivers to take medical decisions. In some cases, they arrive at shared decisions, and in other cases, they experience some conflict, with negative implications on the care process. No systematic collection of “moral conflicts”, i.e., conflicts pertaining to ethically related issues, occurring among patients and their caregivers in cancer care path is available in current bioethics literature.

**Methods:**

Using PRISMA guidelines, we conducted a systematic review of bioethics literature, broadly considered, in five major databases: PubMed, Web of Science™, PsycINFO, Cinahl and Philosopher’s Index. Titles, abstracts and full texts of identified papers were screened for relevance. The snowball technique and citation tracking were used to identify relevant publications. Data analysis and synthesis were conducted in line with the QUAGOL methodology.

**Results:**

Twenty-two publications were included. Publication dates ranged from 1999 to 2021. We distinguished four different types of conflict: “conflict between values”, “conflict between interests”, “conflict related to decisional responsibilities and autonomous decision-making” and “relational conflicts related to existential and/or ontological differences”. Conflict among patients and caregivers can be exacerbated by several factors both related to caregiver’s characteristics, family history and disease.

**Conclusion:**

The evidence collected shows the importance of considering also the ethical dimension of the oncological care process, especially in its decisional component.

**Supplementary Information:**

The online version contains supplementary material available at 10.1007/s00520-025-09548-w.

## Background

During the cancer treatment trajectory, patients may be asked to take several decisions, spanning from the choice of the hospital/care centre to the daily management of cancer treatments’ effect, to a do-not-resuscitate (DNR) order [1, 2]. Bioethical literature has for long emphasised that individuals have the right to act in accordance with a principle of autonomy, which entitles them to decide by themselves and exercise self-determination regarding their own health [3]. However, academic literature has also shown that patients with life-threatening conditions such as cancer are not only particularly vulnerable but also almost always in a network of interpersonal relationships that can influence their decisions, impacting on their autonomy, if interpreted in its traditional, non-relational, meaning [4–8]. Indeed, throughout the disease trajectory, family members often step into the role of caregivers, who provide ordinary support to practical and emotional patient issues, including personal care, medication adherence, assistive schedule integration and management [9–16]. Caregivers may be partners, close or extended family members and even friends [8, 15, 17].

Although the caregiver’s role and her level of involvement and responsibility can vary depending on the needs of the patient and the stage of the disease [18], becoming a caregiver may be unexpected and usually they do not have sufficient prior guidance and preparation. Therefore, caregivers may experience some psychological distress [19–21]. Moreover, quite often are patients themselves who intentionally involve caregivers in the decision-making process; by doing this, caregivers themselves may feel entitled to influence patients’ decisions. This process inevitably brings a series of emotional reactions, interpersonal dynamics and expectations [8, 11, 22–27].

However, it is certainly true that a diagnosis of cancer and its treatment affects both the patient and his/her family, resulting in a family disease [28–30]. Recent research has shown that cancer has the same impact on both the oncological patient and her family in terms of psychological distress, even altering the family structure and role [23, 28, 31]. A cancer diagnosis may indeed pose various challenges and tensions within the family, and, as suggested by the Family System Illness model (FS), the caregivers’ involvement may be both a potential source of conflict and a valuable resource [32]. In other words, the presence of the caregiver may be both a facilitator and a barrier to the decision-making process [22, 23, 31]. In particular, it has been shown that agreement among patients and caregivers on the decisions to be made may reduce patient’s “decision regret” and improve adherence to care process [23, 33, 34]. Conversely, disagreement among patients and caregivers may have a negative impact on the patient, affecting the understanding of medical information, compliance with therapies and, consequently, the patient’s quality of life, including his/her relationship with the caregiver [35–39].

Conflictual decision-making processes can arise not only when the (competent) patient and the caregiver are not aligned on the decision to be made, but also when the caregiver disagrees with the decision taken by an incompetent patient at the time when he/she was competent (e.g., before losing the decision-making capacity). However, conflicts within a family may be somehow considered a natural phenomenon that not necessarily ends up in a disruptive event. Differently, if generator of mutual dialogue and perceived as functional, family conflict may also turn into something positive [12, 35].

The aim of this systematic review is to investigate decisional conflicts arising in cancer patients’ care process, originating from caregiver’s involvement, as well their outcomes on the dyadic relationship and the care process. Although there may be several perspectives through which this phenomenon may be analysed, such conflict will be here explored through the lens of bioethical scrutiny, namely, as an ethical concept.

Although several studies have explored this phenomenon from a psychological perspective (i.e., psychological conflicts occurring in the patient-caregiver relationships) [40–43], to the best of our knowledge no systematic collection of *moral conflicts between patients and caregivers* is available in current literature. A “moral conflict” may be defined as a conflict of evaluation and/or decision, occurring between (at least) two subjects (in this case patients and caregivers) who evaluate the same scenario (e.g., a therapeutic proposal) through different moral perspectives. Therefore, a conflict becomes a moral conflict when the agents disagree on the decision to be taken (e.g., which therapeutic option to follow) based on the fact that the two agents endorse different (sometimes conflicting) moral values.

Filling this scholarly gap appears of utmost importance not only to shed some light on a still underexplored research perspective, but also to have a comprehensive view of the cancer patient care process, including the fundamental component of the relationship with caregivers. Therefore, better clarifying the origin, nature, impact and potential solutions to moral conflicts occurring among the patients-caregivers’ dyads appears, to us, as a further step towards the improvement of the cancer patient condition.

## Materials and methods

In order to have a comprehensive overview of potential moral conflicts originating from caregiver’s involvement during the oncological care process, we conducted a systematic review of bioethics literature, broadly considered, thus including also applied philosophy and medical anthropology literature.

The review process consisted of the following steps: first, we identified our research questions; second, we defined thematic groups; third, on the basis of the thematic groups, we developed research strings to be inserted in databases which were then queried; then, results were all screened according to the process described in the PRISMA guidelines^1^ [44, 45]. The whole process is described in detail below.

### Research questions

This systematic review aims to provide an answer to the following groups of research questions:

1. Are there some moral conflicts, i.e., conflicts related to *ethically related issues*, occurring among patients and their caregivers within cancer care path? If yes, what is the origin and nature of these moral conflicts?
2. What are the triggers of (or factors leading to) moral conflicts occurring in the oncological care process? What is the impact of moral conflicts on patients and caregivers?
3. What are the solutions proposed by the literature to moral conflicts occurring in the oncological care process?

### Search strategy

The afore research questions were then drawn out in four groups of concepts to systematise our literature research (Table 1).

**Table 1:** Group of organizing concepts and associated database search terms

Group 1: caregiver	Group 2: involvement	Group 3: cancer	Group 4: ethics
Caregiver—Caregivers Attendant Parent—Parents Relative—Relatives Partner Spouse Significant others Caretaker Companion	Involvement Relationship Agreement Disagreement Engagement Support Participation	Cancer Cancer patient Cancer treatment Cancer path Cancer trajectory Oncology Oncological patient Oncological treatment Oncological path Palliative care End of life Decision-making	Ethics Bioethics Medical anthropology Ethical issue Ethical challenge Bioethical issue Bioethical challenge Moral dilemma

The purpose of Group 1 was to gather scientific papers focusing on the concept of caregiving and/or akin concepts, e.g., caregiver, spouse, parent. The purpose of Group 2 was to collect scientific papers focusing on the concept of involvement and/or akin concepts, e.g., relationship and the concept of conflict and/or akin concepts, e.g., disagreement. The purpose of Group 3 was to select a specific population of investigation, i.e., cancer patients. The purpose of Group 4 was to define the disciplinary domains where to find conceptualizations of the terms belonging to Group 2. As mentioned in the Background, the concept of conflict may be analysed from different perspectives: clinical, psychological, bioethical, philosophical, anthropological, etc. We narrowed to publications exploring this issue from a bioethical perspective, broadly considered, thus also including applied philosophy and medical anthropology. Some publications raised considerations at the crossroads of applied ethics and clinical psychology. In case of a thematic overlap between these two disciplinary domains, publications were eventually included, to be sure to include contributions potentially relevant from a bioethical perspective.

Each group concept was expressed in specific database search terms in a suitable format for the different database queries (Table 2). Research strings were developed by the first author (CC) in consultation with the co-first author (VS).

**Table 2:** Search strings

**Database**	**Date**	**Group 1: family**		**Group 2: involvement**		**Group 3: cancer**		**Group 4: ethics**	**Results**
PubMed	10 March 2021	((((((((((((((caregivers[MeSH Terms]) OR (spouses[MeSH Terms])) OR (parents[MeSH Terms])) OR (companion[MeSH Terms])) OR (caregiver[Title/Abstract])) OR (caregivers[Title/Abstract])) OR (attendant[Title/Abstract])) OR (spouse[Title/Abstract])) OR (parents[Title/Abstract])) OR (parent[Title/Abstract])) OR (relatives[Title/Abstract])) OR (partner[Title/Abstract])) OR (significant others[Title/Abstract])) OR (companion[Title/Abstract])) OR (caretaker[Title/Abstract])	AND	((((((((((((consensus[MeSH Terms]) OR (family conflicts[MeSH Terms])) OR (social support[MeSH Terms])) OR (involvement[Title/Abstract])) OR (relationship[Title/Abstract])) OR (engagement[Title/Abstract])) OR (agreement[Title/Abstract])) OR (consensus[Title/Abstract])) OR (disagreement[Title/Abstract])) OR (family conflicts[Title/Abstract])) OR (support[Title/Abstract])) OR (social support[Title/Abstract])) OR (participation[Title/Abstract])	AND	(((((((((((((((((((neoplasm[MeSH Terms]) OR (palliative care[MeSH Terms])) OR (terminal care[MeSH Terms])) OR (decision making[MeSH Terms])) OR (decision making, shared[MeSH Terms])) OR (cancer[Title/Abstract])) OR (neoplasm[Title/Abstract])) OR (cancer patient[Title/Abstract])) OR (cancer treatment[Title/Abstract])) OR (cancer path[Title/Abstract])) OR (cancer trajectory[Title/Abstract])) OR (oncology[Title/Abstract])) OR (oncological path[Title/Abstract])) OR (oncological treatment[Title/Abstract])) OR (oncological patient[Title/Abstract])) OR (palliative care[Title/Abstract])) OR (end of life[Title/Abstract])) OR (terminal care[Title/Abstract])) OR (decision making[Title/Abstract])) OR (shared decision making[Title/Abstract])	AND	(((((((ethics[MeSH Terms]) OR (bioethics[MeSH Terms])) OR (anthropology, medical[MeSH Terms])) OR (ethics[Title/Abstract])) OR (bioethics[Title/Abstract])) OR (anthropology, medical[Title/Abstract])) OR (ethical challenge*[Title/Abstract])) OR (bioethical challenge*[Title/Abstract])	*n* = 1494
Web of Science	10 March 2021	TS = (caregiver* OR spouse* OR parents OR companion OR attendant OR relatives OR partner OR significant others OR caretaker)	AND	TS = (consensus OR family conflicts OR social support OR involvement OR relationship OR engagement OR agreement OR disagreement OR support OR participation)	AND	TS = (neoplasm OR palliative care OR terminal care OR decision making OR shared decision making OR cancer OR cancer patient OR cancer treatment OR cancer path OR cancer trajectory OR oncology OR oncological path OR oncological treatment OR oncological patient OR end of life)	AND	TS = (ethics OR bioethics OR medical anthropology OR ethical challenge* OR bioethical challenge*)	*n* = 1697
PsycINFO	10 March 2021	(caregivers or spouses or parents or companion).mh. or caregivers.ti. or caregivers.ab. or attendant.ti. or attendant.ab. or spouse.ti. or spouse.ab. or parents.ti. or parents.ab. or relatives.ti. or relatives.ab. or partner.ti. or partner.ab. or significant others.ti. or significant others.ab. or companion.ti. or companion.ab. or caretaker.ti. or caretaker.ab	AND	(consensus or family conflicts or social support).mh. or involvement.ab. or involvement.ti. or relationship.ab. or relationship.ti. or engagement.ab. or engagement.ti. or agreement.ab. or agreement.ti. or consensus.ab. or consensus.ti. or disagreement.ab. or disagreement.ti. or family conflicts.ab. or family conflicts.ti. or support.ab. or support.ti. or social support.ab. or social support.ti. or participation.ab. or participation.ti	AND	(neoplasm or palliative care or terminal care or decision making or shared decision making).mh. or cancer.ab. or cancer.ti. or neoplasm.ab. or neoplasm.ti. or cancer patient.ab. or cancer patient.ti. or cancer treatment.ab. or cancer treatment.ti. or cancer path.ab. or cancer path.ti. or cancer trajectory.ab. or cancer trajectory.ti. or oncology.ab. or oncology.ti. or oncological path.ab. or oncologica path.ti. or oncological treatment.ab. or oncological treatment.ti. or oncological patient.ti. or oncological patient.ab. or palliative care.ti. or palliative care.ab. or end of life.ti. or end of life.ab. or terminal care.ti. or terminal care.ab. or decision making.ti. or decision making.ab. or shared decision making.ti. or shared decision making.ab	AND	(ethics or bioethics or medical anthropology).mh. or ethics.ti. or ethics.ab. or bioethics.ti. or bioethics.ab. or medical anthropology.ti. or medical anthropology.ab. or ethical challenge*.ti. or ethical challenge*.ab. or bioethical challenge*.ti. or bioethical challenge*.ab	*n* = 154
Philosopher’s Index	10 March 2021	noft(caregiver*) OR noft(spouse*) OR noft(parents) OR noft(companion) OR noft(attendant) OR noft(relatives) OR noft(partner) OR noft(significant others) OR noft(caretaker)	AND	noft(consensus) OR noft(family conflict) OR noft(social support) OR noft(involvement) OR noft(relationship) OR noft(engagement) OR noft(agreement) OR noft(disagreement) OR noft(support) OR noft(participation)	AND	noft(neoplasm) OR noft(palliative care) OR noft(terminal care) OR noft(decision making) OR noft(shared decision making) OR noft(cancer) OR noft(cancer patient) OR noft(cancer treatment) OR noft(cancer path) OR noft(cancer trajectory) OR noft(oncology) OR noft(oncological path) OR noft(oncological treatment) OR noft(oncological patient) OR noft(end of life)	AND	noft(ethics) OR noft(bioethics) OR noft(medical anthropology) OR noft(ethical challenge*) OR noft(bioethical challenge*)	*n* = 220
Cinahl	10 March 2021	(MH caregivers or MH spouses or MH parents or MH companion or TI caregiver or AB caregiver or TI caregivers or AB caregivers or TI attendant or AB attendant or TI spouse or AB spouse or TI parents or AB parents or TI parent or AB parent or TI relatives or AB relatives or TI partner or AB partner or TI significant others or AB significant others or TI companion or AB companion or TI caretaker or AB caretaker)	AND	(MH consensus or MH family conflicts or MH social support or TI involvement or AB involvement or TI relationship or AB relationship or TI engagement or AB engagement or TI agreement or AB agreement or TI consensus or AB consensus or TI disagreement or AB disagreement or TI family conflicts or AB family conflicts or TI support or AB support or TI social support or AB social support or TI participation or AB participation)	AND	(MH neoplasm or MH palliative care or MH terminal care or MH decision making or MH shared decision making or TI cancer or AB cancer or TI neoplasm or AB neoplasm or TI cancer patient or AB cancer patient or TI cancer treatment or AB cancer treatment or TI cancer path or AB cancer path or TI cancer trajectory or AB cancer trajectory or TI oncology or AB oncology or TI oncological path or AB oncological path or TI oncological treatment or AB oncological treatment or TI oncological patient or AB oncological patient or TI palliative care or AB palliative care or TI end of life or AB end of life or TI terminal care or AB terminal care or TI decision making or AB decision making or TI shared decision making or AB shared decision making)	AND	(MH ethics or MH bioethics or MH medical anthropology or TI ethics or AB ethics or TI bioethics or AB bioethics or TI medical anthropology or AB medical anthropology or TI ethical challenge* or AB ethical challenge* or TI bioethical challenge* or AB bioethical challenge*)	*n* = 370
Total									3935

Five major databases were queried: PubMed, Web of Science, PsycINFO, Philosopher’s Index, Cinahl. These databases cover the fields of ethics, bioethics, philosophy and medical anthropology.

Research was conducted on the 10th of March 2021, using only a language filter restriction. Table 2 shows not only the terms used to make the search, but also the number of results returned using the search terms.

Using EndNote (version X9, Clarivate Analytics, Philadelphia, PA, USA) reference library, resulting citations of the identified papers were merged and duplicates (*N* = 866) were manually deleted by the first author (CC). Then, titles, abstracts and full texts of identified papers were screened according to the inclusion and exclusion criteria (see the section Inclusion and Exclusion criteria below). Abstract screening (*N* = 580) was performed independently by the first and co-first authors (CC and VS) to verify the consistency of our criteria and to ensure scientific and methodological rigorousness of the abstract selection. In 91.53% of the abstracts (*N* = 540 out of 580), the authors agreed to include them for the next step. The remaining abstracts (8.47%, corresponding to 40 articles) were subjected to discussion until an agreement was reached. Then, the first and co-first authors (CC and VS) screened the full text of the remaining records (*N* = 60) independently. A total of 17 articles were included in the review process. In case of unavailable papers, the authors contacted the first and/or corresponding author to request a PDF copy of it. Snowball technique and citation tracking were also used to identify potentially additional relevant publications: six additional articles that met the inclusion criteria were retrieved through reference manual searching and included.

**Fig. 1:**
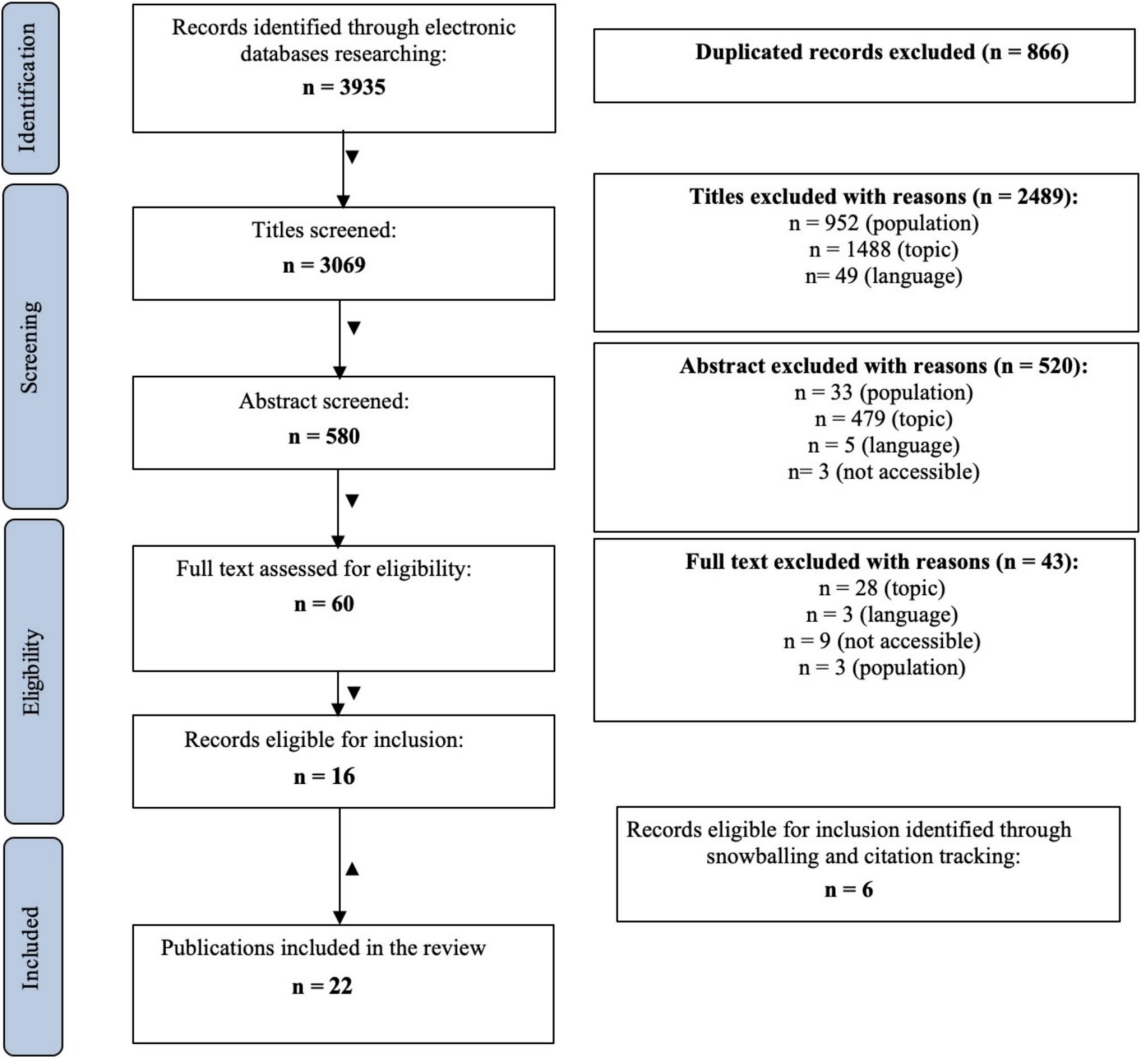
PRISMA flowchart

Finally, a total of 22 studies were included in the review. The search process was conducted according to the statement and flowchart of the Preferred Reporting Items for Systematic Reviews and Meta-Analysis (PRISMA) [45] (Fig. 1).

The final list of included publications is reported in Table 3.

**Table 3:** Final list of included publications

*N*°	Author	First author’s publication country	Publication year	First author’s background
[35]	Benson, J. J.,	USA	2019	Family gerontology
[22]	Blackler, L	USA	2016	Bioethics
[46]	Eliott, J.,	Australia	2008	Social scientist
[29]	Hansen, H. P	Denmark	2020	Anthropology
[47]	Hauke, D	Germany	2011	Internal medicine
[48]	Kagawa‐Singer, M	USA	2003	Nursing and anthropology
[49]	Korfage, I. J	UK/Netherland	2013	Economy and epidemiology
[24]	Laryionava, K	Germany	2021	Oncology
[12]	Laryionava, K	Germany	2018	Oncology
[50]	Levine, C	USA	1999	Psychology
[5]	Levine, C	USA	2000	Psychology
[51]	Mazer, B. L	USA	2014	Anatomic and clinical pathology
[6]	Osamor, P. E	USA	2018	Medical sociology
[7]	Sahlberg-Blom, E	Sweden	2000	Nursing
[8]	Ullrich, A	Germany	2020	Medical psychology
[36]	François, K	Australia	2017	Life science and public health
[37]	Hamano, J	Japan	2018	Internal medicine
[52]	Kramer, B.J	USA	2009	Qualitative social research
[53]	Kramer, B.J	USA	2013	Qualitative social research
[38]	Hopeck, P	USA	2017	Communication
[54]	Boelk, A.Z	USA	2012	Sociology and social work
[55]	Vivian, R	UK	2006	Nursing

### Inclusion criteria

Publications were included based on the following conditions: (a) they address the concept of conflict resulting from caregiver’s involvement in the cancer patient’s care process as specific topic; (b) they include only legally-competent patients (i.e., patients considered capable of decision-making capacity), *or* patients who had expressed their own perspective when still competent; (c) the conflict was a moral conflict, broadly considered (see Background); (d) research articles were published in English.

### Exclusion criteria

The following publications were excluded from the review: (a) studies addressing the caregiver’s involvement in the oncological care path of the competent patient, but which do not present conflict as specific topic of investigation; (b) studies focusing on the concept of conflict generated by caregiver’s involvement, but not specific to the oncological context; (c) studies focusing on the concept of conflict generated by caregiver’s involvement in the oncological context, occurring among competent patients and their caregivers, but where conflict is not a moral conflict (e.g., as purely psychological phaenomenon, etc.); (d) contributions not published in English, (e) editorials, books, conference proceeding and book chapters.

### Data extraction and synthesis

Data analysis and synthesis were conducted according to the five preparatory steps of the coding process detailed in the “Qualitative Analysis Guide of Leuven” (QUAGOL) [56]. Initially, the first author (CC) read twice all the included articles, underling the relevant parts for the purpose of the review. Subsequently, the same author summarised narratively these parts and created a conceptual scheme for each publication (an example of the conceptual scheme is available in Supplementary Information). Then, the first (CC) and the co-first author (VS) examined each conceptual scheme to assess its accuracy in relation to the included publications, and, in case of doubt, discussed the content of the conceptual schemes until they agreed on their adequacy. Finally, conceptual schemes were analysed in relation to our research questions. This led to a comprehensive scheme that integrated the most relevant ethical issues emerging from caregiver’s involvement during the oncological care process of competent patients (e.g., types of ethical conflicts, nature of these conflicts). This scheme was controlled against previous QUAGOL steps to ensure its adequacy. Finally, we synthesised a description of the results.

## Results

### General description of included publications

Twenty-two publications met our inclusion criteria and were therefore included in our systematic review. A detailed description of the general characteristics of the included publications is reported in Table 4.

**Table 4:** Description of characteristics of included publications

**Analysed features (number of publication)/paper *N* as listed in **Table 3
*Type of research* • Theoretical: 22, 5, 6, 55 • Qualitative: 35, 46, 29, 49, 24, 12, 50, 51, 7, 8, 36, 53, 38, 54 • Quantitative: 47, 37 • Mixed methods: 48, 53
*Participants enrolled* • Patients: 22, 46, 29, 47, 48, 49, 51, 7, 55 • Caregivers: 35, 46, 47, 51, 8, 36, 37, 54, 55 • Healthcare professional: 49, 24, 12, 50, 51, 36, 53, 38, 54, 55
*First author’s publication country* • Americas: 35, 22, 48, 5, 51, 6, 7, 53, 38, 54, 55 • Europe: 29, 47, 49, 24, 12, 50, 8, 36, 37 • Australia: 46 • Asia: 52
*Years of publication* • 2021–2016: 35, 22, 29, 49, 12, 50, 7, 36, 37, 52, 54 • 2015–2011: 47, 24, 6, 38, 55 • 2010–2006: 46, 53 • 2005–2001: 48 • Before 2001: 5, 51, 8

Because of the very specific focus of the topic investigated, we included publications covering theoretical, qualitative and quantitative literature giving different ethical perspectives regarding a conflictual involvement of caregivers during the oncological care path of competent patients. Of the twenty-two articles included in the review, fourteen conducted a qualitative evaluation of the concepts through interviews [7, 8, 12, 24, 29, 35, 36, 38, 46, 49–52, 54], and four articles described theoretical models for family conflict management [5, 6, 22, 55], two of which made a qualitative evaluation through a case report [22, 55]. The remaining papers used mixed methods [48, 53], and only two were quantitative studies [37, 47].

Most of the publications were published between 2016 and 2021 [6, 8, 12, 22, 24, 29, 35–38], and the others covered a period up to 1999. Studies were from the Americas, Europe, Australia and Asia; that is, the first author and/or corresponding author was from one of these continents. However, most of the papers came from studies conducted in the Americas (*N* = 11) and in Europe (*N* = 9).

The enrolled participants were healthcare professionals in five studies [12, 24, 38, 50, 53], caregivers (e.g., family members) in three studies [8, 35, 37] and patients in four studies [7, 22, 29, 48]. In the remaining six studies, participants included both patients and caregivers [46, 47], or patients and healthcare professionals [49] or patients, caregivers and healthcare professionals [51, 55]. Only in three studies, patients were not included as participants [36, 54]. No sample was present in the three theoretical studies [5, 6, 52].

Our analysis and synthesis supported a threefold structure of the included publications (Table 5). This structure can be conceived of as sections. The first section outlines the different theoretical conceptualizations of the broadly defined concept of “moral conflict”. For each conceptualization, the conflict is analysed mostly considering the perspective of the two members of the dyad, patients and caregivers. In case where patient-caregiver conflict was referred to as originating from (or strictly related to) physician’s involvement, we also analysed the triadic relationship among the patient, his/her caregiver and the caring physician (mostly the oncologist). The second section presents the factors associated with conflicts occurring among patients and caregivers. The third and last section deals with the strategies proposed by the literature that could be used to address such conflicts.

**Table 5:** Global scheme emerging from analysis of the 22 included publications*

Main results	Included articles
*1. Caregivers identity*	
Relatives	35, 47, 24, 7, 8, 36, 52
Children	35
Spouse/partners	35, 22, 29, 48, 6, 8
Family members	22, 46, 49, 12, 50, 5, 37, 52, 38, 54, 55
Friends	8
*2. Type of conflict*	
Conflict between values	35, 47, 49, 24, 12, 50, 36, 52, 53, 38, 54
Conflict between interests	35, 22, 47, 24, 12, 50, 5, 51, 7
Conflict related to decisional responsibilities	35, 22, 46, 47, 12, 6, 7, 8, 37, 55
Relational conflicts	29, 48
*3. Factors related to conflict*	
Caregiver’s stressors	35, 22, 24, 12, 5, 8
Familial context	12, 5, 37, 52, 53, 54
Disease duration and/or severity	47, 50, 54
*4. Conflict management*	
Formal support provider (e.g., psycho-oncologist, clinical ethics consultant/clinical ethics support services)	35, 22, 49, 12, 5, 36
Co-determination	6, 7
Psychological techniques (e.g., assertive communication, emotional self-care, refocusing, reconciling, referring, reflecting, reframing)	35, 38

*A single article can be represented more than once

### “Conflicts” among patients and caregivers in the care process: labels, meanings and object under discussion

In the included articles, what we referred to as “conflict” is labelled differently and presents different connotations, sometimes overlapping. We identified four types of moral conflicts, which we will refer herein to as “conflict between values” or “ethical disagreement” [12, 24, 35, 36, 38, 47, 49, 50, 52–54], “conflict between interests” [5, 12, 22, 24, 35, 47, 50, 51], “conflict related to decisional responsibilities and autonomous decision-making” [6–8, 12, 22, 35, 37, 46, 47, 55] and “relational conflicts related to existential and/or ontological differences” [29, 48]. To provide a better conceptualization of our results, we will present these types of conflicts one after the other. However, in the included publications, these are sometimes presented as overlapping, sometimes defined as occurring at the same time. Moreover, as we will explain below, different types of conflicts may occur in the different stages of cancer disease (see Table 6).

**Table 6:** Type of conflict in relation to disease’s stage

Type of conflict	Stage of disease		
	**Stages 0, I, II, III**	**Stage IV and/or advanced cancer**	**End of life**
Conflict between values		24	35, 47, 49, 12, 50, 36, 52, 53, 38, 54
Conflict between interests	5	2, 24, 5, 51	35, 47, 12, 50, 5, 7
Conflict related to decisional responsibilities	6	22, 6, 8, 37	35, 46, 47, 12, 6, 7, 55
Relational conflicts	29, 48		

#### Conflict between values

In some articles, the term conflict has been interpreted as conflict between values [24, 35, 36, 38, 47, 49, 50, 52–54]. It refers to the disagreement between the patient and her caregiver on the decision to be taken, that originates from the *different values and/or preferences* endorsed by the members of the dyad, practically operationalised in the decisional options [24, 35, 38, 47, 49, 50, 52–54]. In other words, in some cases, the conflict between patient and caregiver arises because the two would opt in favour of highly different (decisional) options, on the basis of value-based reasons (i.e., different moral values underlying the different decisional options). This phaenomenon has been extensively investigated in bioethics literature, under the label of “ethical disagreement”. The conflict between values, in the oncological care path, may regard decisions concerning treatment goals, whether to opt in favour of formal caregiving or bring the patient into a hospice, discharge or leave the patient hospitalised, move the patient to formal care facilities and decide who should provide care, especially at the end of patient’s life [24, 35, 36, 38, 47, 49, 50, 52, 54]. These conflicts are framed by the parties as discussion, arguments, discord or expressed dissent and may emerge during consultations with clinicians [35, 49, 52]. In this case, the conflict between values may become both an obstacle to the patient’s care path or provide useful information to the clinician who can intervene and manage the conflict [12, 24, 49].

Sometimes, conflict between values may also occur between the patient-caregiver dyad and the medical team [47, 50, 52]. Examples of the latter regard the inadequacy of service resources, unprofessional behaviour of the staff, lack of proper communication on advanced care planning [36].

#### Conflict between interests

Moral conflict may arise when patients or caregivers ground their decisions in personal interests, creating what we refer to here as “conflict between interests” [5, 7, 12, 22, 24, 35, 37, 47, 50, 51, 55]. In this case, conflict does not result from the opposition between genuinely endorsed moral perspectives, but rather from the clash of partisan interests, at least by one side of the dyad, more often caregivers. Sometimes, caregivers consider their own and familial interest above that of the patient, because patient’s best interest may be not always in line with the interests of other family members, thus creating a form of hidden conflict [5, 12, 22, 35, 37, 50, 51, 55]. In other cases, faced with the idea of losing a beloved one, caregivers may cling to patients in advanced stages of their disease, desperately demanding that all steps be taken to prolong life regardless of the patient’s wishes and real-life prospects [22, 24]. Some caregivers were even found to act against patients’ known or assumed wishes/preferences [47]. This happened because of several reasons: caregivers felt the economic or care burden, they did not accept patients’ critical situation or simply because they were too intimately involved in relationship with the patient to find it very hard to let her go [12, 22, 35, 50]. Although it does not appear appropriate to speak of conflict between values in these scenarios, the former is sometimes invoked to cover conflicts based on more strictly individualistic reasons.

#### Conflict related to decisional responsibilities and autonomous decision-making

Some authors show that conflicts among patients and caregivers can be also related to decisional responsibilities. These may originate from the non-recognition of the other as entitled of (but not obliged to) autonomous decision-making, thus misinterpreting what it means respecting the other’s decisional autonomy. Throughout the oncological care path, patients and caregivers are agents that may be asked to make medical decisions in autonomy. An autonomous individual is a person with the power to make her own decisions, speak or act without the interference from others [6, 7, 22, 35, 55]. However, in daily oncological practice, two problems may occur. On the one hand, it may happen that, albeit ideally fully entailed to take the decision, patients experience a compromission of their autonomy, because of caregivers’ interferences [22, 35, 47]. On the other hand, some patients may decide to give up their autonomous choices, completely relying on their caregivers. With respect to this second scenario, while in some cases caregivers willingly take on this role, in other circumstances, caregivers may perceive such a decisional responsibility as a burden. This means that they feel obliged to exercise their decision-making capacity in contexts where they feel they would not be necessarily required to do so [35, 46].

In our included publications, the concept of autonomy does not only emerge as a topic of overt conflict, but also as an underlying source and/or consequence of other conflicts [35]. Most people make decisions influenced by a complex network of social relationships [6]. This happens also to patients, even if throughout the care pathway they struggle to maintain their autonomy, or to recover it, if missing [35]. This is experienced as a direct threat to the caregiver’s autonomy, especially when the decision puts the patient at risk or increases caregiver’s practical responsibilities, by delaying or prolonging their commitment [35]. In other cases, conflicts over autonomy are about patients’ reluctance to assume responsibility over their own care decisions [7, 35, 46]. This may happen especially at the end of life when the medical decisions to be made may involve cardiopulmonary resuscitation (CPR) and consequently the order do-not-resuscitate (DNR) [46]. This decision is experienced by patients and caregivers as a decision regarding patient’s life and death. In making these decisions, patients and caregivers implicitly raise moral judgments about the value of the patient’s life and her relationship with significant others. Insofar as decisions in this context may be perceived as deeply related to personal (moral) integrity, this can lead patients and caregivers experiencing frustration both during the decision-making process and once the decision is made [46].

In some cases, patient’s autonomy could be compromised if they are (unintentionally) exposed by their families to undue influence, coercion and manipulation, all resulting in pushing them taking medical decisions that are not in line with previously held values, beliefs or perspectives [6, 12, 22]. Family pressure or coercion occurs through verbal threats, harassment, reprimand, intimidation or other manipulative tactics designed to force vulnerable patients to change established beliefs or preferences [22]. This most often happens to patients with a history of inequality of power, changes in family roles and relationship status and progressive illness [12, 22]. When family members do not accept the patient’s condition, they may ask for more aggressive treatments which may even go against the treatment goals advocated by the medical team [12]. Patients with compromised autonomy due to caregiver coercion may eventually comply with caregiver decisions but only to avoid conflict or protect the family. This way, patients may experience moral distress [12, 22]. Similarly, when caregivers violate what may be considered as values of “good” caregiving, patient autonomy and will, integrity and honesty, they experience moral suffering, namely, painful feelings of self-blame, insecurity and personal disappointment or disappointment in others [8]. Sometimes the patient’s compromission of autonomy may be seen during the medical consultations. Indeed, sometimes caregivers speak or answer to the physician’s questions as if the patient were not in the room or unable to speak for him/herself, making claims about the patient's views [51]. Conflicts over autonomy also occurred among caregivers and the extended family. In this situation, primary caregivers felt themselves threatened and disheartened by the family in their role [35]. During the patient’s care pathway, the caregiver may experience the need to self-govern her personal life and to return to activities and relationships that they had suspended or neglected during caregiving. Even these cases may be interpreted as compromissions of autonomy [35].

#### Relational conflicts related to existential and/or ontological differences

In other cases, conflicts concern relational aspects, namely, aspects genuinely related to (and originating from) the relationship between the members of the dyad. According to this account, conflicts may originate from the failure to recognise the individuality of the other, which may lead to a rupture in their social relationship [48]. In this case, the other is not acknowledged as different from the subject, namely, as a separate entity [48]. Not acknowledging the other as a separate being, with her own feeling, experiences and perspectives, has an in impact on intersubjectivity [29]. Some authors consider it also in contrast with the virtue of “compassion”, i.e., the ability to be aware of the emotions of others, to better understand how they feel and their desires and to harmony [29, 48]. In other words, although cancer patients tend to appreciate caregivers’ support, it may occur that they do not feel understood or acknowledged in their own individuality [29]. This issue is framed differently in different contributions. In some cases, caregivers are defined as unable to fully understand the needs and requirements of patients, because they have never had cancer, or because they are unable to meet patients’ needs without burdening patients [29]. In other cases, male patients, frustrated by the loss of strength due to cancer, may come into more conflict with their wives [29, 48]. Differences in expectations regarding the behaviour of the other may generate some conflict within the dyads: the efforts of the caregivers are not always positively experienced by patients [29, 48].

Although some degree of conflict is generally present in social relationships, some authors believe that, at least in some cases, this may be so disruptive to impact on the structure of the relationship itself, thus leading to specific disputes, challenges, breakups and violence [29]. The terms “friction” and “dissonance” are used in our selected papers precisely in relation to the kind of conflict that appears to destroy the social relationship [29].

However, not all relational conflicts are perceived negatively. Indeed, disagreement could be the symptom of a still existing relationship. When the dyad disagrees on the treatment, this shows that the dyadic relationship still exists and that cancer may be addressed as a shared problem.

### Triggers of and/or factors related to patients-caregivers’ dyads conflict

Our included publications report that conflicts between patients and caregivers about care decisions may be exacerbated by several factors. On the one hand, during the oncological care process, caregivers may feel overwhelmed by the situation and can experience emotional and moral distress, burden, anxiety and depression [12, 22, 24, 35]. Moral distress consists of a painful feeling or psychological vulnerability caused by the inability to follow what is perceived and/or believed to be the right ethical course of actions, or to act according to one’s own values due to internal or external constraints [8, 22]. In our publications, moral distress and burden correlate with feelings of depression, helplessness, exhaustion, frustration, resentment, guilt and self-accusation [8, 35]. Such a burden may lead the caregiver to argue with his/her patient. Indeed, caregiver stressors—such as confusion, miscommunication, frustration, resentment, fear and sadness—proved to have an impact on decision-making, causing some conflicts within the family nucleus [22, 24, 35]. In some cases, a feeling of resentment emerged in caregivers who perceived a reduction in their autonomy and a lack of compassion and trust on the part of the patient or other family members [35]. Indeed, the resentment experienced by caregivers was often also brought about by the involvement of a third party, such as a member of the extended family who did not support emotionally and practically the caregiver, for instance, allowing them no relief, denying them the possibility to share their feelings, or accepting sharing some of the caregiver’s labour [35]. Moreover, usually, caregivers have their own private life outside the patient to take charge of, and sometimes, they have to act as a bridge with other family members; frustrations also originate in conditions where time for themselves was limited by the patient’s care and needs, making it impossible for the caregivers to pursue their hobbies and interests, or to spend quality time with their own family [5, 8, 35]. Feelings of frustration arise in the context of difficult decision-making, or, differently, when there was a lack of decision-making options, i.e., when there was no further open therapeutic chance potentially offered to the patient [8]. In this case, however, this cannot be configured specifically as a moral conflict, but it was the result of the caregivers’ frustration with an uncertain care process, or uncertain outcome, or lack of support. Caregivers affected by the aforementioned feelings (e.g., resentment, frustration, burden) may experience a great deal of “cognitive dissonance”, namely, the state of discomfort felt when two or more modes of thought contradict each other [35]. Indeed, caregivers may have contradictory feelings about the care they did (or did not) deliver and about their attitudes towards their role, thus experiencing psychological distress [35]. Caregivers may experience feelings of guilt and regret, struggling between their desire for more personal autonomy and that of being more devoted caregivers [35]. Frustration and guilt may also be experienced by patients, especially in context of end-life decisions [46].

Moreover, family context may be also an increasing factor for conflicts. Our publications showed that the younger age of the family, the assertiveness of family members in decision-making for patient care, and the limitations in communication among family members, are significantly associated with increased conflict [5, 12, 37, 52, 53]. Also, historical relationship patterns, i.e., how families interacted before the onset of the disease, family structure and family socio-economic conditions, may influence how family members approach each other during the oncological care process [5, 12, 52, 54]. Moreover, caregivers are often surrounded by the so-called “extended family”, i.e., any family members beyond the nuclear family (e.g., grandparents, cousins, aunts, uncles). In some cases, the extended family may appear unsupportive and non-collaborative; this may increase the burden of caregivers, while also creating a conflictual environment [35].

Finally, in one publication reporting results from a quantitative study, a statistically significant tendency was found between conflict and duration and/or severity of the disease: in presence of prolonged disease and/or diseases severity, conflicts appeared enhanced [47, 50, 54].

### Conflict management strategies

Our selected articles present several strategies for conflict management. First, caregivers may manage the conflict by involving a formal support provider (i.e., a hospice staff member, a psycho-oncologist, even a clinical ethicist) both as a mediator to help caregivers communicating with the patient and the other members of the family, or to take on some of the caregiving responsibilities directly [22, 35, 36, 38, 49]. To resolve conflicts, formal support providers can use some strategies, most of which belong originally to the psychological field. A first proposed strategy is “refocusing” both with the caregiver alone and with all family members; this consists in helping families to consider what would be the best actions for the patient, also remembering that the patient is the one who is entitled to take the final decision. Refocusing allowed families to focus their attention on the patient, thus downsizing family wishes. Another strategy is “reconciling”, namely, a formal support provider which may help caregivers and patients reconciling past grievances between family members and the patient. In this case, it is not necessary to work together with the caregiver and the patient, but it may be sufficient to work with the members of the dyad individually. The third most common strategy reported by formal support providers is “referring”, that is, rely on others who are part of their professional network (e.g., sending caregiver, patient or both to other professionals depending on their needs). The last two strategies identified in selected publications are “reframing”, namely, a technique which aims to help family members to understand the patient’s medical condition by using simple words, and “reflecting”, i.e., a strategy that enables a formal support provider to understand the impact of medical decisions on the two members of the dyad, to ensure the family is informed and supported. By focusing on listening patients and caregivers, the formal support provider may be able to understand caregivers and patients’ needs and adapt their responses to these needs. All these strategies are not self-excluding and therefore can be combined one with the other [38]. In other cases, conflict was “resolved” once caregivers realised and accepted that some degrees of conflict were part of the oncological care path and of the relational differences set above. Therefore, the mere acceptance of the inevitability of some conflict was, in some cases, a driver for its resolution [35].

Other times, a solution to decision-making conflicts reported in the included publications was the active involvement of both members of the dyad, patients and caregivers, resulting in a process defined “co-determination”. This strategy, mostly widespread across clinical ethicists, means that the patient actively takes part in the decision-making process, considering not only her own wishes and needs but also those of her caregiver; the resulting decision is actually shared between the two [6, 7].

Finally, some caregivers may cope with the conflictual environment showing high resilience during medical decision-making, using direct and assertive communication, namely, affirming their needs and seeking formal support, emotional self-care through meditation, positive self-talk, cognitive restructuring, or engaging in positive internal dialogue [35]. Thoughtful conversations among caregivers and patients about their different opinions and wishes allowed them to resolve the conflict, because caregivers and patients may become aware of each others’ views and, in some cases, agreed to disagree [22].

## Discussion

The aim of this review was to provide a systematic collection of moral conflicts occurring among patients and their caregivers in cancer care path, still absent in current literature. We analysed relevant publications that appeared from 1999 to 2021 in the bioethics literature, asking a threefold question: (i) are there some moral conflicts occurring among patients and their caregivers in cancer care path? If yes, what is the origin and nature of these moral conflicts? (ii) What are the triggers of (and/or factors leading to) moral conflicts occurring in the cancer care process? What is the impact of moral conflicts on patients and caregivers? (iii) What are the solutions proposed by the literature to moral conflicts occurring in the cancer care process?

The main findings are discussed in depth in the following paragraphs, with the aim of gaining a better understanding of how to properly deal with caregiver’s involvement.

From our systematic review, it emerges that patients are almost always accompanied by at least one caregiver along the oncological care path [7, 8, 22, 24, 29, 35, 37, 47–49, 51], and this is in line with previous findings. If considered together with the interpretation of cancer as a family disease [28–30], this leads to the consideration that a comprehensive approach to cancer treatment requires considering not only patients but also their caregivers and particularly their relationship. In general, caregivers appear to help patients throughout the oncological care process; however, sometimes they also appear to interfere in the decision-making process, creating some issues for the patients themselves. Therefore, it appears of utmost importance understanding how to involve caregivers in the oncological care trajectory.

Regarding the first research question, our systematic review provides further evidence to the fact that cancer patients and their caregivers can experience episodes of conflicts or tensions about care decisions and treatment goals [5–8, 12, 22, 24, 29, 35–38, 46–55]. As extensively shown in the Result section, such conflict presents different meanings and connotations, sometimes overlapping. We distinguished four different types of conflicts. In some cases, conflict originates from a disagreement on the decision to be taken, that is, from the *different values and/or preferences* endorsed by the members of the dyad (“conflict between values”) [24, 35, 47, 49, 50, 52–54]. In other cases, conflict arises when the patient or the caregiver ground her decisions in personal interests (“conflict between interests”) [5, 12, 22, 50]. Otherwise, conflicts may emerge due to the non-recognition of the other as entitled of (but not obliged to) autonomous decision-making and consequently to a compromission of their respective autonomy (“conflict related to decisional responsibilities and autonomous decision-making”) [6, 12, 22, 35]. Finally, conflict may originate from the failure to recognise the individuality of others which leads to a rupture in the social relationship (“relational conflicts related to existential and/or ontological differences”) [29, 48].

The main finding in this respect is that conflict—at least in the oncological context—does not have only a clinical or psychological connotation, but it may also present an ethical connotation, deserving devoted consideration and analysis. Since in these cases conflict presents a multilayer dimension, the proper way to approach it is not from a single disciplinary perspective. Differently, it requires the synergistic collaboration of different disciplines able to capture and analyse the conflict from different angles, thus allowing a multidisciplinary approach to the problem. From a practical standpoint, these considerations corroborate the idea that the gold standard is establishing multidisciplinary teams composed, at least, by the devoted clinicians (e.g., the medical oncologist, the surgeon, the radiotherapist, the palliativist), the psycho-oncologist, and the clinical ethicist. And, if the tendency nowadays is to approach complex oncological cases involving not only relevant clinicians but also psycho-oncologists [57–61], clinical ethicists working shoulder to shoulder healthcare professionals are still virtuous exceptions. Regarding this issue, it may be here useful to recall that the so-called Clinical Ethics Support Services, in the form of single consultants working within multidisciplinary teams or Clinical Ethics Committees supporting, when necessary, healthcare professionals, are a rapidly growing reality, already implemented in several countries, especially the US, UK, France and The Netherlands [58, 62–67]. In line with current literature [68], our systematic review provides additional evidence on the importance to find strategies to further implement Clinical Ethics Support Services in those realities where they are still missing, particularly in the context of cancer care.

As to the second research question (factors related to conflict and impact of such conflict on patients and caregivers), from our analysis, it emerges that conflict among patients and caregivers can be exacerbated by caregiver’s distress or relational patterns [12, 22, 24, 35, 37, 52, 54]. Accordingly, it is important to explore caregivers’ background (i.e., religion, culture and ethnicity) and mood, since this information may help reducing conflict [36, 50, 54]. Regardless of conflict’s reasons and with respect to its impact on the care process, our included articles show that conflict can both be an obstacle to the patient’s care path and provide useful information, depending on how conflict is approached by the different parties [12, 24, 49]. As reported, the conflict within the dyad is not always perceived negatively, since it can be the symptom of a still existing relationship. When the dyad disagrees on patient’s care treatment, this shows that the dyadic relationship still exists and that cancer may be considered a shared problem, that has to be addressed jointly and as part of the relational process itself [12, 35, 48]. In other words, “silencing” the conflict does not always appear as the best option. Indeed, as shown by recent literature, taking moral conflicts seriously and "educating" them so as to make them functional has been correlated with a lowering of moral distress [69].

This finding, which leads us to the third research question, is in line with the idea that the gold standard in medical decisions is the so-called "shared decision-making" among patients, physicians and, in some cases, caregivers [4–8, 23, 31, 46, 70–76]. Shared decision-making is defined as the decision-making process which is purposed to arrive at a truly shared solution; in this case, it is the decision that both members of the dyad agree on or consent to. In the latter case, although patients and caregivers may have different initial opinions, after proper discussion, one of the members of the dyad may decide to embrace the other's viewpoint. By doing so, he/she is not precluding himself/herself from the exercise of self-determination. In other words, shared decision-making and autonomous decision-making should not be considered mutually excluding approaches. The member of the dyad who freely decides to make this movement in the direction of the other is still exercising self-determination. The expression “shared decision-making” points also to the fact that what is shared is not only the final decision, but also the decisional process, which takes the form of a dialogue between interlocutors. Interpreted through the lens of bioethics scrutiny, shared decision-making is the dialogical approach which, theoretically, embeds a principle of “relational autonomy” and, from a practical standpoint, may end up resulting in a process of co-determination [4, 77]. A relational understanding of autonomy focuses on the importance of the social reality around the individual in taking decisions. Accordingly, relational accounts of autonomy consider it as first a particularistic and contextual feature, conversely rejecting the idea that autonomy should be only a theoretical and acontextual principle [22, 78–82]. In this context, relational autonomy possesses also additional traits, already pointed out by contemporary literature. Interpreted in line with shared decision-making, relational autonomy means first of all promoting *inclusiveness* in the decisional process, therefore including the perspectives of patients and caregivers and—if possible—also of caring physicians [83–85]. Moreover, relational autonomy may be also considered as a *gradual* and *dynamic* principle, rather than an all-or-nothing concept [86–89], which may be expressed along a continuum and whose value and impact may vary depending on the specific context, decision and care process [83, 86, 87, 90–95]. This is also in line with bioethics literature which has considered autonomy as an important but not overriding value. In the oncological context, for instance, compassion [84, 90], hope [96] and empathy [84, 86, 88, 97] appear of utmost importance. Also empirical studies have shown that decision-making processes based only on the individual exercise of autonomy are not in line with patients’ preferences, especially in the end of life context [1, 12, 98, 99]. Narrowing to the oncological field, cancer patients prefer to share decisions with their physicians and/or caregivers and, in some specific cases, even delegate them to decide [7]. Indeed, although some patients wish to have full control of the decision-making process, others prefer to defer decision-making to family members or, at least, considering their interests, trying to incorporate these interests in the final decision. In these scenarios, also known as “joint decision-making” [6] and/or “co-determination” [7, 100, 101], patients actively participate in the decision-making process understanding their medical condition, discussing it with family members and doctors, listening to their opinions, beliefs and perspectives and ideally arriving at a joint decision. This does not necessarily mean that, after the dialogical process, decisional actors will surely agree on the content of the decision; it may also be that caregivers and/or healthcare professionals come to accept the patient’s will, considering the latter as the option in line with patient’s best interest.

Related to conflict management’ strategies, the authors find important to observe that at least some of the strategies identified in our articles are far from being routinary practice. Although shared decision-making is currently considered the gold standard in contemporary medicine, healthcare systems tend to be exposed to conditions that drive towards efficiency, which make it difficult to implement this practice routinely [102, 103]. Moreover, though clinical ethicists are quite widespread in US and in some European countries, the wide implementation of Clinical Ethics Support Services in every cancer centre is far from being a reality. This means that potential moral conflicts arising in clinical practice run the risk of not being identified and/or adequately addressed, due to a lack of the designated figure to properly deal with them. Finally, with regard to psychological conflict management strategies, several considerations should be pointed out. First, psycho-oncological support services are not present in all cancer centres. This may appear critical insofar as psychological conflict management strategies are not easily used by formal support providers other than psychologists. Also, these strategies are actually used only when social support is requested by patients or caregivers, where generally the request for psychological support by the side of caregivers is very rare. Finally, mediation between patients and caregivers is hardly implemented in the hospital setting unless explicitly requested.

In conclusion, our findings seem to corroborate the idea that self-determination as a non-relational concept may appear as rather inadequate in the oncological field, where, differently, shared decision-making and relational autonomy have been shown as more appropriate. Indeed, from a diagnosis of cancer and its subsequent care process till the end-of-life, cancer has a very high impact both on the patient and her caregiver, and on the extended family more generally. As we tried to show, within the cancer care pathway, both the concept of moral conflict and the potential solutions to it are deeply relational: the conflict, since it arises from the dyadic relationship between two actors, patients and caregivers, who experience the oncological disease as affecting both parties; the latter, since solutions to conflicts require recovering a relational account of autonomy, where the patient is the final actor, but the caregiver turns out to be a major player in the cancer patient’s care process, both directly and indirectly. However, as reported above, there is still an important gap from the ideal to the reality of conflict management in cancer care. Shared decision-making, the implementation of Clinical Ethics support services and even devoted psycho-oncological support services are still rare practices [104, 105]. Though too demanding and refined strategies for conflict management may be counterproductive, increasing rather than lowering psychological burden and moral distress of patients and caregivers [69], nonetheless fostering a productive conversation, embedding, if possible, patient’s preferences in cancer trajectory and promoting caregivers’ participation should not be considered additional tasks for oncologists, but fundamental practices related to the very essence of the care process.

### Strengths and limitations

The main strength of this systematic review is that, although focused on moral conflicts, it has been conducted and written by a senior bioethicist (VS) together with a clinical psychologist (CC). This allowed us to have a broader picture on the very complex issue of moral conflict, which, in some cases, appears at the crossroads of medicine, ethics and psychology. This multidisciplinarity also reflects the complexity of cancer disease and cancer patient’s care. Moreover, our systematic review presents a high methodological robustness, not only in data extraction, but also in data analysis and synthesis, due to the QUAGOL methodology employed.

As to potential limitation, this review collected articles published till 2021. Although the topic of moral conflict between patients and caregivers in cancer contexts may be considered a niche topic, nonetheless, it may be the case that more recent publications on the topic are not covered by this review.

## Conclusion

The aim of this systematic review was to investigate the concept of moral conflict originating from caregiver’s involvement along the oncological care process, which represents a still undertheorised issue in current bioethics debates. To gain a comprehensive overview of this topic, we probed the literature about the origin and nature of moral conflicts, factors leading to, impact of and solutions to moral conflicts occurring during the oncological care trajectory.

Our analysis resulted in a taxonomy composed of four types of moral conflicts, i.e., conflicts about ethically related issues, broadly conceived. Our analysis also showed that moral conflict may be further exacerbated by factors related to the caregiver’s condition (e.g., psychological and moral distress, demographic characteristics), the family context (e.g., relational pattern), but also the entity of the disease (i.e., duration and severity). Several strategies have been proposed in the literature to overcome moral conflicts, which span from psychological to ethical support services. Altogether, the evidence collected shows the importance of considering also the ethical dimension of the (oncological) care process, especially in its decisional component.

## Supplementary Information

Below is the link to the electronic supplementary material.

ESM 1 (DOCX 25.1 KB)

## Data Availability

All data generated or analyzed during this study are included in this published article in Tables 3, 4, 5.

## Abbreviations

CPR: Cardiopulmonary resuscitation
DNR: Do-not-resuscitate
FS: Family System Illness model
PRISMA: Preferred Reporting Items for Systematic Reviews and Meta-Analyses
QUAGOL: Qualitative Analysis Guide of Leuven
UK: United Kingdom
US: United States

## Footnotes

1 A PRISMA guidelines statement specifically designed for ethics literature was recently published (PRISMA-Ethics – Reporting Guideline for Systematic Reviews on Ethics Literature: development, explanations and examples [44]. However, since the search for this systematic review was done prior to the publication of this article, we followed the process described in Liberati et al. [45].
