# Supplementary material for: Moral conflicts among patients-caregivers dyads in oncological care pathway: a systematic review of the ethical literature
Source: Support Care Cancer. 2025 Jun 6;33(7):548. doi: 10.1007/s00520-025-09548-w (PMC12141141; doi:10.1007/s00520-025-09548-w)
Supplement: Supplementary file 1 — (DOCX 25.1 KB) [file 520_2025_9548_MOESM1_ESM.docx]

| **2** | **Blackler, L. (2016). Compromised autonomy: when families pressure patients to change their wishes. *Journal of Hospice & Palliative Nursing*, *18*(4), 184-191.** |
| --- | --- |

| **SYNOPSIS** | |
| --- | --- |
| Focus | Patient/Family disagreement (conflict) |
| Definition/  Conceptualization of Conflict | Conflict between patient and caregivers (or family members) emerges when there is disagreement between them about treatment/therapeutic decisions. This conflict can be exacerbated as cancer advances and the efficacy of treatment is less certain, because medical decisions may involve cardiopulmonary resuscitation (CPR) and, consequently, do-not-resuscitate (DNR) orders, which are experienced as decisions concerning the patient's life and death. In making these decisions, patients and caregivers implicitly raise moral judgments about the value of the patient's life. For example, patients may wish to sign the DNR order based on their own definition of quality of life, while caregivers and family members, faced with the idea of losing a loved one, may desperately demand that all measures be taken to prolong life, regardless of the patient's wishes and actual life prospects. In this case, there is a conflict between the wishes of the patient and the caregiver, based on personal moral values and self-interest. Moreover, when the caregiver forces the patient to change his or her mind, there is an impairment of the patient's decision-making autonomy that evolves into a conflict. |
| Theoretical Approaches | Relational autonomy |
| Population | 78 years old man diagnosed with widely metastatic gastrointestinal cancer and his wife |
| Type of paper | Qualitative (case report from a clinical ethics consultation) |

**Conceptualization of patient/family disagreement (conflict)**

Cancer patients and their caregivers may experience episodes of conflicts or tensions about care decisions and treatment goals. As cancer advances and the efficacy of treatment is less certain, these conflicts may be exacerbated due to severity of the medical decisions, that may involve cardiopulmonary resuscitation (CPR) and, consequently, do-not-resuscitate (DNR) orders. In making these decisions, patients and caregivers may experience conflict that can be explored through the lens of (bio)ethical scrutiny. In particular, patients and caregivers may implicitly raise moral judgments about the value of the patient's life. For example, patients may wish to sign the DNR order based on their own definition of quality of life, demanding that living intubated is a life not worth living. Instead, caregivers and family members, faced with the idea of losing a loved one, may desperately demand that all measures be taken to prolong life, regardless of the patient's wishes and actual life prospects. In this case, there is a conflict between the preferences and values of the patient and the caregiver, but also due to different personal interest. Moreover, when the caregiver forces the patient to change his or her mind, there is an impairment of the patient's decision-making autonomy that evolves into a conflict. Caregivers seems to non-recognize the patient as entitled of autonomous decision-making.

**Theoretical framework**

Respect for autonomy is a fundamental principle in bioethics, especially regarding decision-making in oncology. However, empirical studies show that decision-making focused solely on the individual exercise of autonomy does not reflect clinical reality and does not align with patient preferences. Indeed, the caregiver’s involvement, and more generally the family’s involvement, in decision-making has been widely documented. This has led to the need for a more contextualized approach that responds to the real-life complexities experienced in oncology. In this regard, the concept of "relational autonomy" may be an appropriate alternative approach. This normative ethical theory was developed primarily by feminists, which rejects classical Kantian conception of autonomy, according to which people are separate autonomous agents independent from social relations. Instead, relational autonomy holds that personal identity is built partially by the social relations (to which family belongs). Indeed, patients are embedded in social relationships especially with their family, who advocate for and assist patients with decisions. In this context, patients may rely on the family member for guidance and support. According to an ethics of care, the patient's final decision is the result of a process consisting of a dynamic and constant dialogue among family members, in which all members consider the preferences, opinions, values and interests of all members. Furthermore, cancer can be perceived as a family disease, leading stressful and challenging time for all family members. Finally, according to the relational autonomy all decisions are influenced by historical, cultural, social, class, race, gender, and familial contexts.

The idea of relational autonomy is also supported by recent research in cognitive psychology, suggesting that individuals make decisions not by themselves but rather in interaction with others and emphasizing the concept of shared decision-making. Shared patient/family decision-making is perfectly acceptable, as long as collaboration is desire. There is concern when family involvement becomes excessive, or is not requested by the patient, or imposes decisions that are inconsistent with the patient's established wishes/desire/value/preference, evolving into conflict.
